# Comparison of visfatin levels in patients with breast cancer and endometrial cancer with healthy individuals: A systematic review and meta‐analysis

**DOI:** 10.1002/hsr2.895

**Published:** 2022-11-18

**Authors:** Hori Ghaneialvar, Samira Shiri, Azra Kenarkoohi, Zahra Fallah Vastani, Alireza Ahmadi, Ali Khorshidi, Roghayeh Khooz

**Affiliations:** ^1^ Biotechnology and Medicinal Plants Research Center Ilam University of Medical Sciences Ilam Iran; ^2^ Clinical Research Development Centre, Taleghani and Imam Ali Hospital Kermanshah University of Medical Sciences Kermanshah Iran; ^3^ Zoonotic Diseases Research Center Ilam University of Medical Sciences Ilam Iran; ^4^ Student Research Committee, Faculty of Allied Medical Sciences Ilam University of Medical Sciences Ilam Iran; ^5^ Department of Epidemiology, School of Medicine Ilam University of Medical Sciences Ilam Iran

**Keywords:** adipokine, breast cancer (BC), endometrial cancer (EC), neoplasm, nicotinamide phosphoribosyltransferase (NAMPT), visfatin

## Abstract

**Background and aims:**

Endometrial cancer (EC) and breast cancer (BC) are prevalent in women. Visfatin is an adipokine that, in addition to being involved in metabolism and inflammation, may also be interested in carcinogenesis. Visfatin measurement in cancer patients has shown that visfatin levels in cancer patients differed from those in healthy subjects. Various studies have shown that the level of visfatin is increased in people within EC and BC, and this difference has a significant relationship with prognosis.

**Methods:**

A comprehensive search of related articles from PubMed, Scopus, Web of Science, and the Google Scholar database was done by November 2021. Eligible articles measured visfatin levels in patients with breast cancer and EC. After selecting the eligible studies, the data were extracted and analyzed using the random effect method.

**Results:**

Given the effect size and the confidence interval obtained, the total level of visfatin in cancer patients was different from that in healthy individuals, and this difference was statistically significant. However, the difference in visfatin levels in patients with breast cancer was much more significant than in patients with EC compared to the control group.

**Conclusions:**

Due to the significant increase in visfatin levels in these patients, visfatin may be a potential prognostic factor in breast and ECs. Visfatin levels in cancer patients differed from those in healthy subjects, and this difference was also statistically significant (*p*‐values = 0.00). Visfatin levels also differed between breast cancer patients and healthy individuals, which was statistically significant (*p*‐values = 0.00). The difference in visfatin levels between patients with EC and healthy subjects was statistically significant (*p*‐values = 0.047).

## 1 INTRODUCTION

Breast cancer (BC) is one of the most widespread kinds of nonskin malignant neoplasm, showing a growing incidence worldwide.^1^, ^2^, ^3^ BC generally begins with ductal hyperproliferation and develops into benign tumors and/or metastatic carcinomas upon constantly being stimulated by different carcinogenic factors.^4^ This cancer is associated with age, genetic history, hormonal status, lifestyle, and obesity.^1^, ^5^, ^6^ Furthermore, adipocyte‐secreted hormones play a substantial role in developing this cancer.^7^

Endometrial cancer (EC) is the most prevalent gynecologic malignancy.^8^ In postmenopausal women, abnormal uterine bleeding is usually associated with EC.^9^ Metabolic disorders, inflammation, impaired immunity, obesity, and hypertension are considerable risk factors.^2^ Evaluation of endometrial biopsies, endometrial curettage, and hysterectomy specimen can facilitate disease diagnosis.^9^ Postmenopausal women with a mean age of 68 are patients mostly diagnosed with EC. In recent years, the prevalence of EC has been increasing.^10^

Adipose tissue, as an endocrine organ, is involved in immunity and homeostasis.^3^ This tissue secretes adipocytokines such as visfatin, resistin, and leptin, which may be helpful in the prognosis and diagnosis of cancer,^2^, ^3^, ^11^ which can be beneficial for cancer prognosis and diagnosis.^11^

Visfatin was identified in 2005. It is a large 52 kDa protein, with its gene being located on chromosome 7q22.2.^2^ Visfatin is recognized as pre‐B‐cell colony‐enhancing factor 1 (PBEF1) or nicotinamide phosphoribosyl‐transferase (NAMPT).^12^ Tumor epithelial cells secrete visfatin autocrinally. Visfatin affects both normal and neoplastic mammary tissues by endocrine and paracrine mechanisms.^1^ It involves various metabolic pathways within mammalian cells, such as oxidation of fatty acids, growth, apoptosis, and angiogenesis.^12^, ^13^ Some investigations have also reported on its inflammatory effects.^3^ Altered serum visfatin levels are associated with different cancers, including breast, endometrial, gastric, and colon.^13^, ^14^ Therefore, it seems that visfatin can be used as a biomarker for cancers.

This study aimed to evaluate the serum concentration of visfatin in patients with EC and patients with BC in comparison with healthy individuals.

## 2 MATERIALS AND METHOD*S*

### 2.1 Search strategy

We investigated the available articles in PubMed, Scopus, Web of Science, and the Google Scholar databases until November 2021. A combination of the following keywords was used in our searches as follows: (“Visfatin” OR Nicotinamide Phosphoribosyltransferase) AND (“BC” or Breast neoplasm) AND (“EC” OR Endometrial Neoplasm).

### 2.2 Inclusion and exclusion criteria

Articles that measured visfatin levels in patients with breast and EC were included in the study. Review studies, letters, and studies in languages other than English were excluded.

### 2.3 Study selection and data extraction

The search was conducted by two independent reviewers (Ghaneialvar H. and Shiri S.) in duplicate to avoid errors. All articles retrieved by the search strategy based on title and abstract were screened for eligibility. The discrepancies among papers were surmounted by discussion and consensus. Data were collected for each document, including the author's name, year of publication, country, age, the total number of participants, number of healthy controls, number of cases, visfatin level in healthy control, and visfatin levels in patients (Table 1).

**Table 1:** Characteristics of included articles

	Author	Year	Cancer type	Country	Age	Total number of participants	Number of healthy controls	Number of cases	Visfatin levels in controls (ng/ml)	Visfatin levels in cases (ng/mg)
1	Xiao‐Yang Li	2013	Breast cancer	China	ND	348	100	248	37.2	65.6
2	Adel M. A. Assiri	2015	Breast cancer	Saudi Arabia	53.682 (case)	150	68	82	15.57	18.36
					52.25 (control)					
3	Adel M. A. Assiria	2015	Breast cancer	Saudi Arabia	67.4 (case)	199	110	89	15.5	18.36
					66.5 (control)					
4	Chrishani Rodrigo	2017	Breast cancer	Sri Lanka	48.69 (case)	84	42	42	0.16	0.35
					47.55 (control)					
5	Chrishani Rodrigo	2017	Breast cancer	Sri Lanka	48.69 (case)	76	38	38	0.12	0.35
					47.55 (control)					
6	Maria Dalamaga	2012	Breast cancer	Greece	61.5 (case)	206	103	103	43.6	57.9
					62.3 (control)					
7	Tarek M. K. Motawi	2020	Breast cancer	Egypt	37.4 (case)	85	45	40	7.33	15.48
					36.85 (control)					
8	Tarek M. K. Motawi	2020	Breast cancer	Egypt	39.02 (case)	85	45	40	7.33	12.04
					36.85 (control)					
9	Tarek M. K. Motawi	2020	Breast cancer	Egypt	41.13 (case)	85	45	40	7.33	18.68
					36.85 (control)					
10	Aseel Mokdad Hatam Abdulwahed	2020	Breast cancer	Iraq	41–70	30	20	10	2.503	**3.653**
11	Wenyan Tian	2013	Endometrial cancer	China	56.69 (case)	240	120	120	15.02	19.65
					55.80 (control)					
12	TolgayTuyan Ilhan	2015	Endometrial cancer	Turkey	ND	84	42	42	8.1	14.9
13	Zhongmin Wang	2019	Endometrial cancer	China	54.93 (case)	151	53	98	0.51	0.55
					53.97 (control)					

### 2.4 Quality assessment

We assessed the quality of the selected articles using a scoring system based on the modified Newcastle Ottawa Scale (NOS) for case‐control studies. Studies that scored five entered the process of meta‐analysis (15).

### 2.5 Statistical analysis

Heterogeneity between studies was assessed using the Q Cochran test and *I* ^2^ index. Egger's test was used to evaluate publication bias. Random effects model was used to combine the result of different studies. Data were analyzed using STATA software ver. 11. A *p*‐value less than 0.05 is considered statistically significant.

## 3 RESULTS

**Figure 1:**
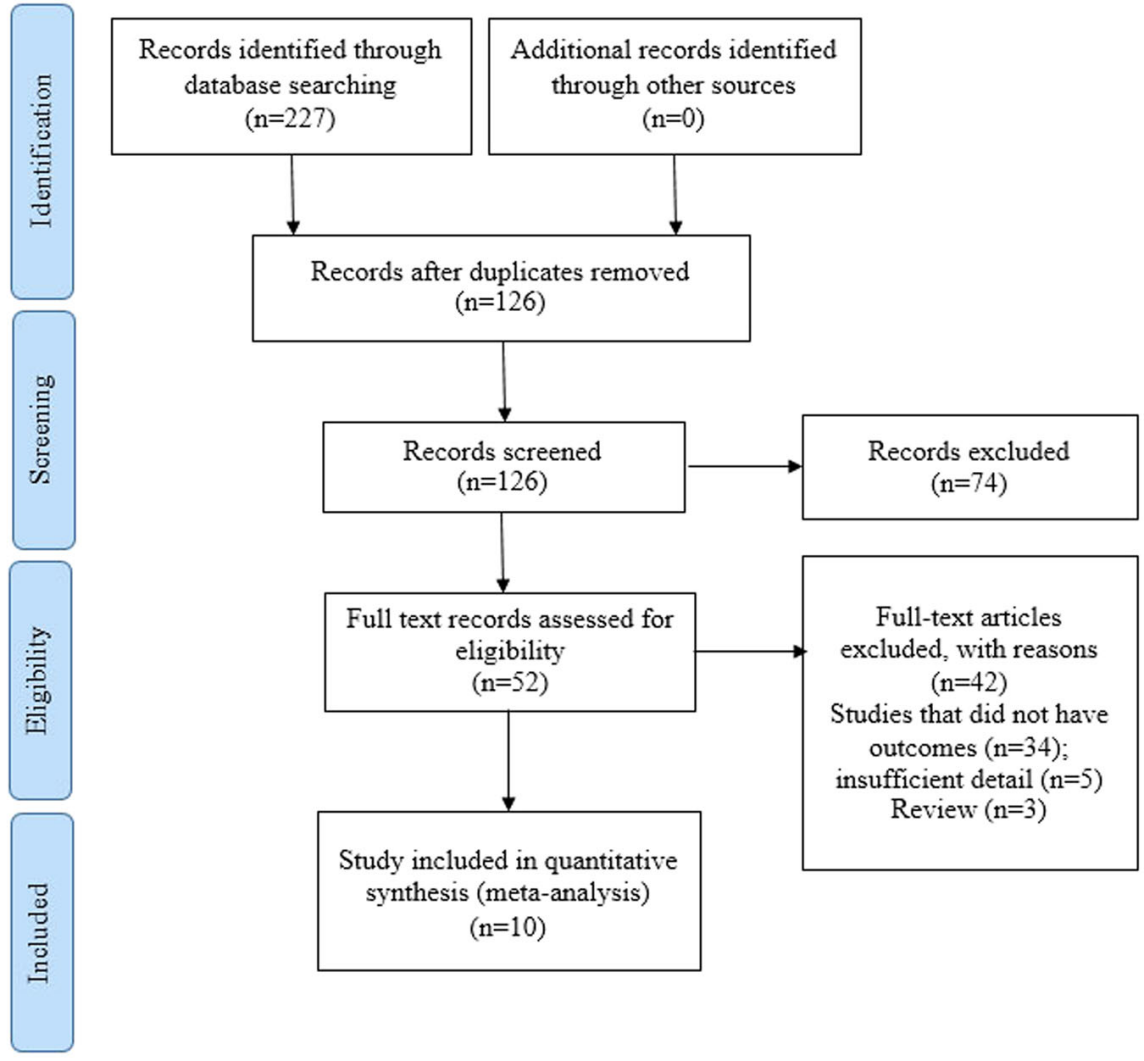
Flowchart eligible studies of endometrial cancer AND breast cancer studies showing the number of citations identified, retrieved and included in the final meta‐analysis

Based on the search strategy, we initially retried 227 articles. Then duplicates were removed, and 126 articles remained. In the next step, the title and abstract of the articles were checked, and 74 papers were excluded. The full text of the remaining 52 articles was evaluated, and 42 were removed due to insufficient information. Finally, 10 articles were included in the meta‐analysis, as shown in Figure 1.

The final sample of this study included 10 articles comprising 732 patients with EC, 260 patients with BC, and 400 healthy individuals.

The heterogeneity in breast and EC studies was 94% and 87%, respectively. Due to the high heterogeneity, a random effect model was used for evaluation.

**Figure 2:**
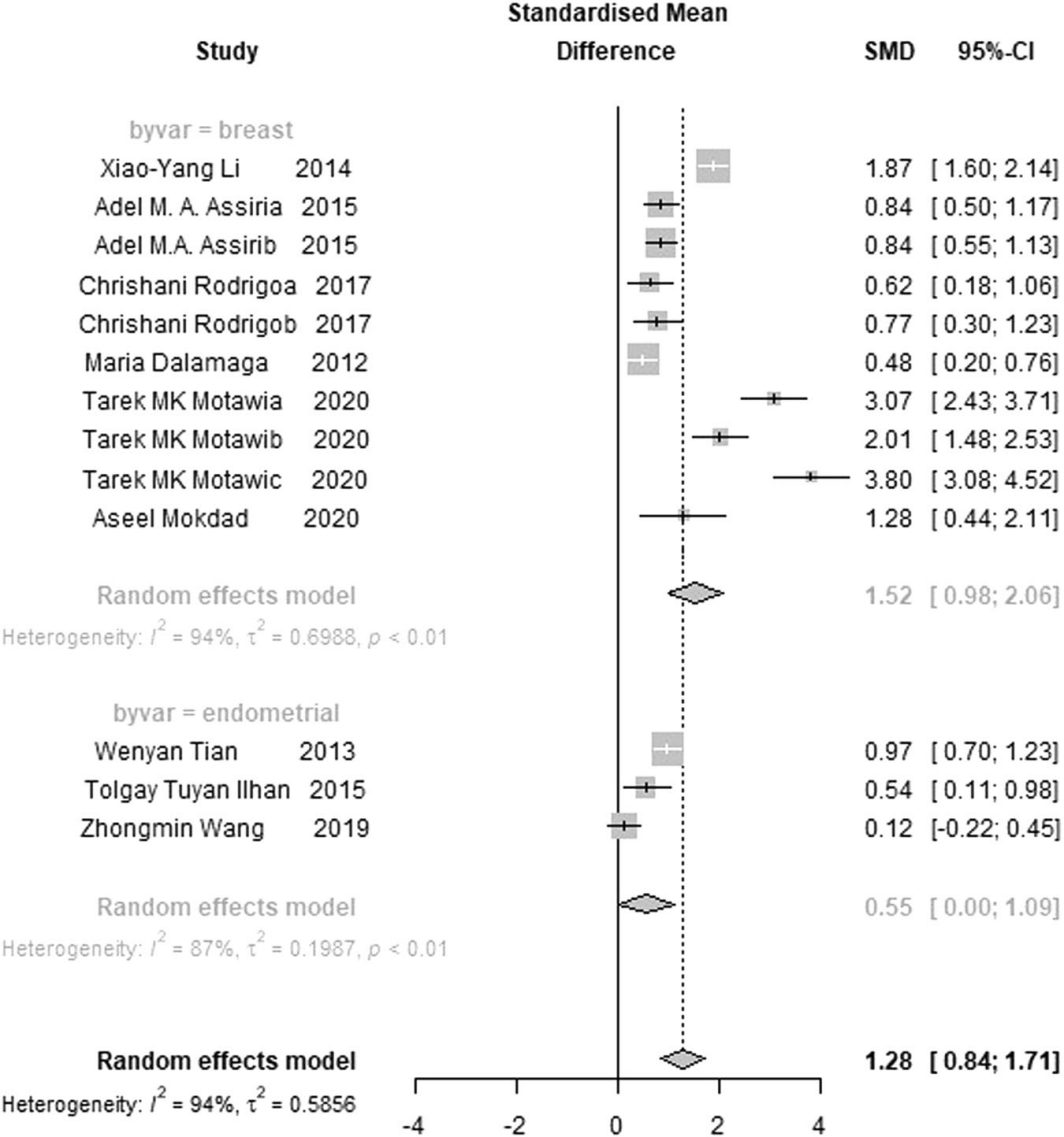
Forest plot: Differences in visfatin levels in the cancer patients and healthy individuals (By cancer: breast and endometrial)

**Figure 3:**
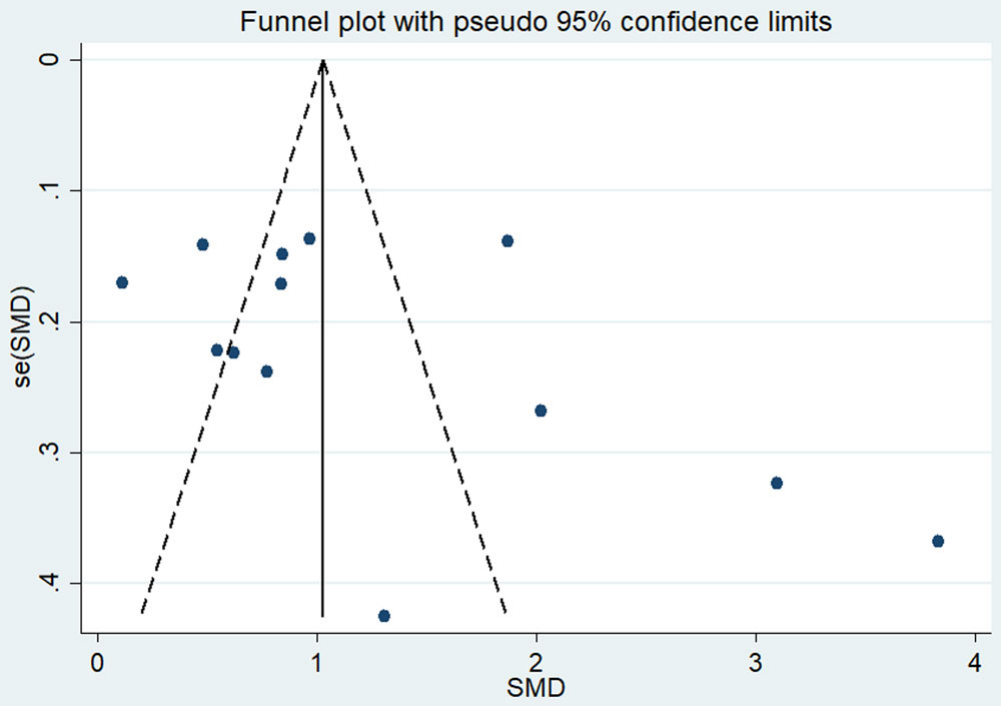
Funnel plot

Visfatin levels in cancer patients differed from those in healthy subjects, and this difference was also statistically significant (*p*‐values = 0.00). Visfatin levels also differed between BC patients and healthy individuals, which was statistically significant (*p*‐values = 0.00). The difference in visfatin levels between patients with EC and healthy subjects was statistically significant (*p*‐values = 0.047), as shown in Figure 2. However, the difference in visfatin levels in patients with BC was much more significant than in patients with EC compared to the control group. The Eger test examined the symmetry of the funnel diagram (Figure 3); the *p*‐value was 0.14. We can conclude that the funnel chart is symmetric. Indeed, these conditions indicated a lack of publication bias (Figure 3).

## 4 DISCUSSION

In this study, results obtained from the analysis of 10 articles showed Visfatin levels in cancer patients differed from those in healthy subjects. In general, elevated serum visfatin levels in people with BC and EC compared to healthy individuals indicate that visfatin may be a promising biomarker for the early detection of such cancers.

Visfatin is an adipokine that, in addition to being involved in metabolism and inflammation, may also be interested in carcinogenesis.^15^, ^16^ Evidence suggests a link between visfatin levels and various cancers.^5^, ^17^, ^18^

One study reported that high levels of circulating visfatin increased the risk of cancer, highlighting the importance of visfatin as a biomarker in the early detection of cancer, especially preventable cancer.^19^ Another study examined the predictive value of visfatin in various cancer types. The results showed that high visfatin expression was an indicator of advanced disease with poor prognostic value.^20^, ^21^, ^22^, ^23^

Studies also suggest an association between serum visfatin levels and tumor growth. Visfatin contributes considerably to the metastasis and synthesis of genes involved in tumor‐associated angiogenesis, like vascular endothelial growth factor, tumor progression, and incursion, such as matrix metalloproteinase in cancers.^24^

In addition, visfatin contributes to the metastatic process in cancers. Visfatin is involved in epithelial‐mesenchymal transmission (EMT) in BC. High levels of visfatin in colorectal cancer affect the chemotherapy of these patients and are associated with a poor response to chemotherapy in this group of patients.^23^

In cancer cell culture, the effect of visfatin on BC cells was investigated; this was evaluated in animal models. Results confirmed the effects of visfatin on tumor growth.^25^

Visfatin causes BC by activating ABL proto‐oncogene 1 (c‐Abl), signal transducer, and activator of transcription 3 (STAT3). Overall, according to the current investigation, serum visfatin levels in patients with BC represented potential predictive values.^26^ Elevated visfatin levels have also been observed in hepatocellular carcinoma patients compared with healthy individuals. Patients with hepatocellular carcinoma with higher circulating visfatin levels also had shorter survival times than those with lower serum visfatin levels.^27^

Visfatin induces malignancy through signaling pathways that contain Rat sarcoma virus (Ras, it belongs to the G‐Small family of proteins), rapidly accelerated fibrosarcoma (Raf), mitogen‐activated protein kinase kinase (MEK1/2), extracellular signal‐regulated kinase (ERK), phosphoinositide 3‐kinase (PI3K), AKT serine/threonine kinase (Akt), and the nuclear factor‐ κB (NF‐κB). In addition, upregulation of the G1‐S phase cell cycle development through upregulating the mRNA levels of cyclin D1 and cyclin‐dependent kinase 2 (CDK2) is caused by visfatin.^26^ In addition, visfatin is involved in cell survival and inhibits cellular apoptosis by tumor necrosis factor alpha (TNF‐α). Thus, studies show that visfatin activates the AKT serine/threonine kinase/phosphoinositide 3‐kinase (AKT/PI3K) and extracellular signal‐regulated kinase/mitogen‐activated protein kinase (ERK/MAPK) pathways, leading to the proliferation of BC cells.^28^

According to the analysis performed in this study, the measured level of visfatin can be used in the control and treatment of breast and EC so that it can be helpful in the early diagnosis of these cancers. Our results show that due to the significant increase in visfatin levels in patients with cancer, visfatin may be a potential prognostic factor in breast and EC.

## AUTHOR CONTRIBUTIONS

**Hori Ghaneialvar**: Conceptualization; investigation; writing–original draft. **Samira Shiri**: Investigation; writing–original draft; writing–review and editing. **Azra Kenarkoohi**: Writing–review and editing. **Zahra Fallah Vastani**: Conceptualization; writing–original draft. **Alireza Ahmadi**: Conceptualization; writing–original draft. **Ali Khorshidi**: Formal analysis; investigation; project administration; software; writing–review and editing. **Roghayeh Khooz**: Conceptualization; investigation.

## CONFLICT OF INTEREST

The authors declare no conflict of interest.

## ETHICS STATEMENT

Ethics approval is waived because this report involves no experiment.

## TRANSPARENCY STATEMENT

The lead author Ali Khorshidi, Roghayeh Khooz affirms that this manuscript is an honest, accurate, and transparent account of the study being reported; that no important aspects of the study have been omitted; and that any discrepancies from the study as planned (and, if relevant, registered) have been explained.

## Data Availability

The data supporting this study's findings are available from the corresponding author upon reasonable request.
